# Estimating the prevalence of obstetric fistula: a systematic review and meta-analysis

**DOI:** 10.1186/1471-2393-13-246

**Published:** 2013-12-30

**Authors:** A J Adler, C Ronsmans, C Calvert, V Filippi

**Affiliations:** 1 London School of Hygiene & Tropical Medicine, Keppel St, WC1E 7HT, London, UK

**Keywords:** Vesicovaginal fistula, Maternal morbidity, Systematic review

## Abstract

**Background:**

Obstetric fistula is a severe condition which has devastating consequences for a woman’s life. The estimation of the burden of fistula at the population level has been impaired by the rarity of diagnosis and the lack of rigorous studies. This study was conducted to determine the prevalence and incidence of fistula in low and middle income countries.

**Methods:**

Six databases were searched, involving two separate searches: one on fistula specifically and one on broader maternal and reproductive morbidities. Studies including estimates of incidence and prevalence of fistula at the population level were included. We conducted meta-analyses of prevalence of fistula among women of reproductive age and the incidence of fistula among recently pregnant women.

**Results:**

Nineteen studies were included in this review. The pooled prevalence in population-based studies was 0.29 (95% CI 0.00, 1.07) fistula per 1000 women of reproductive age in all regions. Separated by region we found 1.57 (95% CI 1.16, 2.06) in sub Saharan Africa and South Asia, 1.60 (95% CI 1.16, 2.10) per 1000 women of reproductive age in sub Saharan Africa and 1.20 (95% CI 0.10, 3.54) per 1000 in South Asia. The pooled incidence was 0.09 (95% CI 0.01, 0.25) per 1000 recently pregnant women.

**Conclusions:**

Our study is the most comprehensive study of the burden of fistula to date. Our findings suggest that the prevalence of fistula is lower than previously reported. The low burden of fistula should not detract from their public health importance, however, given the preventability of the condition, and the devastating consequences of fistula.

## Background

The World Health Organisation defines an obstetric fistula (referred to as fistula in the text below) as an “abnormal opening between a woman’s vagina and bladder and/or rectum through which her urine and/or faeces continually leak [1]”. Classifications of fistula vary, but they generally include fistulae from obstetric causes including vesicovaginal fistula (VVF) and rectovaginal fistula (RVF). Fistulae have devastating consequences [2,3], particularly in low income countries where women have less geographical and financial access to appropriate surgical care for repair. In high income countries they are also devastating, but they are very rare and surgery to repair them occurs more rapidly.

In high income countries, fistulae are due to iatrogenic causes; generally the result of radiation therapy and surgical interventions [4]. In low income countries where access to intrapartum care may be restricted, fistulae are associated with a prolonged or obstructed labour, most commonly occurring when a baby’s head becomes lodged in the mother’s pelvis cutting off blood flow to the surrounding tissues. Prolonged obstruction can cause the tissues to necrotise leading to fistula formation [2].

Women with fistulae often experience horrific or difficult associated conditions which stem either from the fistulae itself or from the prolonged or obstructed labour which caused it [3]. The most obvious consequences are incontinence, either urinary [3], faecal or both. The constant leakage of urine and faeces can also lead to damage to the vulva and thighs [2]. Fistulae are linked with social ostracisation [5] and marginalisation [6]. Many case series show high rates of divorce or separation [7,8], absence of sexual intercourse [6,7], loss of fertility and amenorrhea [9,10] and depression [8,11] among women who have a fistula.

Fistulae are thought to have the highest prevalence where maternal mortality is high, but there is great uncertainty about the actual prevalence [2]. In the 2000 Global Burden of Disease, Dolea and AbouZhar estimated that 0.08% of all births and 2.15% of “neglected obstructed labour births” resulted in fistula [12]. These estimates came from four studies only, all of which were in sub-Saharan Africa and two were hospital-based. In 2006, the WHO estimated that more than 2 million young women throughout the world live with untreated fistula, and that between 50,000 and 100,000 new women are affected each year [1]. These statistics originated from countries’ rapid needs assessments and physician’s reports, mostly available in the grey literature, and not from epidemiological studies using robust design, and almost none include a denominator.

In 2007, Stanton and colleagues wrote a paper [13] on the challenges of quantifying fistula. They described three types of publications reporting on frequency, incidence or prevalence of fistula. The first category of papers relied on secondary and tertiary citations (many of which culminated in personal communications) and reported the number of patients treated without denominators. The second type of publications relied on declarations made by the authors themselves, or on “surgeons’ estimates” but the source of data was unclear. The third type of studies described methods and provided appropriate denominators, but with varying degrees of transparency. Stanton and colleagues were only able to find four papers in this third category [13].

The aim of our review is to provide improved estimates of the prevalence and incidence of fistula by broadening the search and including studies that may previously have been overlooked. Unlike previous reviews of fistula [12-14], we include studies that examine a broad spectrum of reproductive morbidity (including morbidity studies where fistula is but one of many outcomes studied) and/or where no cases of fistula were reported.

## Methods

A two-stage systematic review was conducted in accordance with the STROBE guidelines (http://www.strobe-statement.org/) using free-text and subject headings in Pubmed, Embase, Popline, Lilacs, WHO’s Eastern Mediterranean database, and African Index Medicus published until the end of 2012. The first stage targeted studies specifically reporting on fistula in the title, abstract or subject headings, including search terms such as fistula, vesicovaginal fistula, and VVF. The second stage aimed at identifying additional studies that examined postpartum and reproductive morbidity more broadly, whether or not fistula was specifically mentioned as a pathology. This search included terms such as reproductive morbidity and maternal morbidity. A full search strategy is available upon request. Only English terms were used in the search, but articles were not excluded based on language.

Reference lists were searched for additional articles. Using Web of Science, all relevant articles were subjected to forward citation searching to obtain further articles.

**Figure 1:**
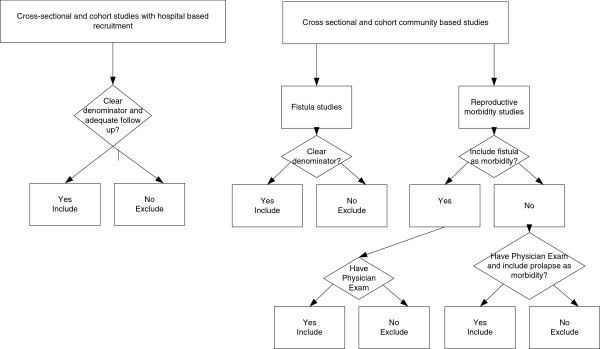
Types of studies included in our analysis.

As illustrated in Figure 1, the following study designs were included: (i) cross-sectional or cohort studies of fistula with hospital based recruitment of pregnant or recently delivered women where the women were followed for and examined at least 30 days after the end of pregnancy and there was a clear denominator; (ii) cross sectional or cohort studies of prevalent or incident cases of fistula in the community and (iii) cross sectional or cohort studies of prevalent or incident cases of reproductive morbidity in the community. Studies were only included if women were examined for the presence of fistula in hospital and/or if a well trained provider performed a physical examination of the genital area. Morbidity studies not reporting or mentioning fistulae but including a robust design, a thorough physical exam of the genital area that reported cases of uterine prolapse were assumed to have zero cases of fistula, as it was assumed that if fistula had been found it would have been reported. Studies relying on women’s self-reports were excluded because self-reports of reproductive morbidity have been shown to be unreliable [13,15,16]. We excluded studies that were conducted before 1990 or published before 1991. We included studies irrespective of sample size but studies without a denominator were excluded. If more than one paper provided results of the same study population, data were first extracted from the article with the greatest amount of information, and supplementary data extracted from the other papers if required.

Data were extracted by a single author (AJA) using a proforma and included information on region, study dates, study population, duration of fistula, type of fistula, risk factors, associated complications and denominators, how fistula were ascertained, sampling technique, number of women and number of deliveries. There are no good tools for looking at study quality in cross-sectional studies, so study quality was assessed using a modified Ottawa-Newcastle score (http://www.ohri.ca/programs/clinical_epidemiology/oxford.asp).

The studies reported on two types of populations of women: women of reproductive age and women with a recent pregnancy. In studies targeting women of reproductive age we calculated the prevalence of fistula per 1000 women of reproductive age. In studies where women were followed after end of pregnancy, we calculated the incidence of fistula per 1000 recently pregnant women (assuming that women were unlikely to have had fistula before getting pregnant). In all cases 95% confidence intervals were calculated using the binomial exact method.

Meta-analyses were conducted using the metaprop command from the R 2.15.3 package meta using a random-effects model [17]. Meta-analyses were conducted summarising the prevalence of fistula among women of reproductive age and the incidence of fistula in recently pregnant women. All studies were stratified by continent and by recruitment in hospital or in the community.

## Results

**Figure 2:**
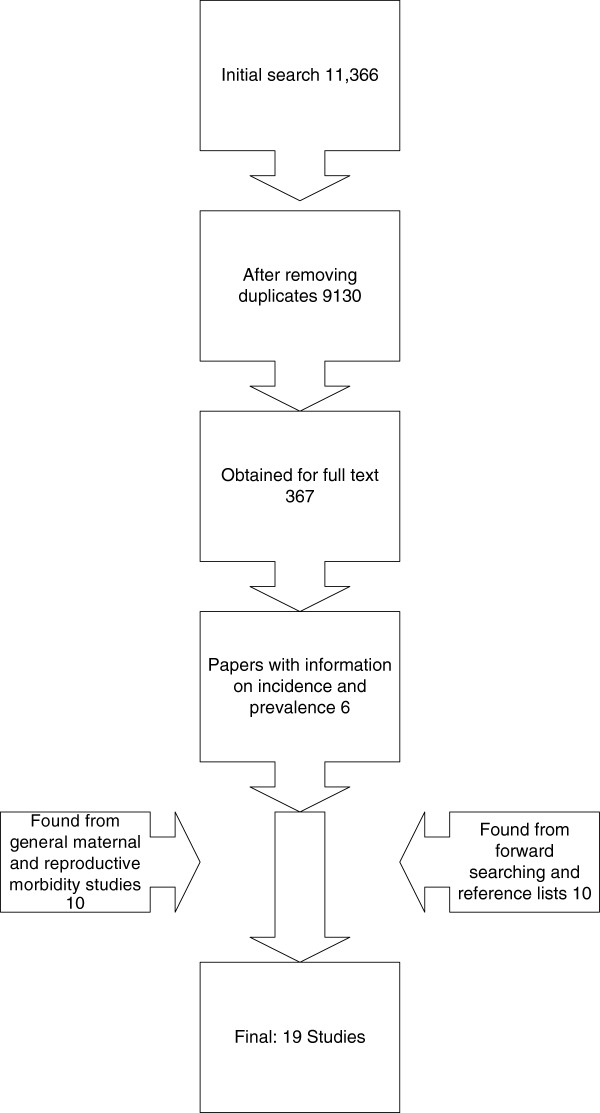
PRISMA diagram of studies.

The initial search of studies focussing on fistula found 9130 references after de-duplication, 367 of which were retained after title and abstract screening. From the 367 articles, six were found to have information on either prevalence or incidence of fistula [18-23]. A further 13 studies [24-34] were found from searching general maternal and reproductive morbidity studies (including three from reference lists and forward citation searching [34-36]) (Figure 2).

Of the 19 studies, 13 were community-based; seven of which reported on fistula [18-20,22,24,33,35] and six did not mention fistula anywhere in the paper. Ten community-based studies reported the prevalence of fistula among women of reproductive age (Table 1) and three reported the incidence among recently pregnant women (Table 2). Six studies recruited women in hospital and followed them for 30 days or more after the end of pregnancy (Table 2). Three studies [21,23,36], report the prevalence of fistula among women after having fistula repairs. These studies were included because it was possible to understand the population that these women came from, and have a denominator (Table 1). The other three studies reported the incidence of fistula among women after giving birth or miscarrying in hospital. These studies recruited women with obstetric complications as well as a sample of women without complications [30-32].

**Table 1:** Characteristics of studies reporting fistula prevalence included in the review

**Author**	**Study area**	**Study design**	**Assessment of fistula**	**Number of fistula**	**Number of women/pregnancies**	**Prevalence(per 1000 WRA)**
Community based studies						
Muleta et al., 2008 [18]	Seven rural administrative regions in Ethiopia	Cross-sectional survey of obstetric fistula	Women reporting leakage of urine, faeces or both examined in the health facilities	44 (untreated)	19,153	1.62 (1.53, 2.64)
Walraven et al., 2001 [24]	Random sample of 20 rural villages in Farafenni, The Gambia	Census of all women aged 15-54 for reproductive morbidity	External, vaginal speculum and bimanual pelvic examination by female gynaecologist	1	1,038	0.95 (0.02, 5.26)
Kulkarni, 2007 [35]	Six PHC areas (urban and rural) in Maharashtra, India	Cross sectional survey of non-pregnant, ever married women with proven fertility for reproductive morbidity	Clinical examination but unspecified what or by whom	1	1,167	0.86 (0.02, 4.8)
Bhatia et al., 1997 [19]	Villages (25% urban, 75% rural) with at least 500 people in Karnataka, India	Cross sectional study of all eligible women under 35 with a child under 5 for reproductive morbidity	External, vaginal speculum and bimanual pelvic examination by female gynaecologist	1	385	2.6 (0.07, 14.39)
Younis et al., 1993 [29]	Two rural villages in Giza, Egypt	Cross sectional study of reproductive morbidity in ever-married, non pregnant women.	Speculum and bimanual examination by female physicians [1]	0	509	0.0 (0.0 , 7.90)
Deeb et al., 2003 [27]	Nabi Sheet, Lebanon	Cross sectional study of reproductive morbidity in ever married, non-pregnant women	Thorough inspection of external genitalia, with speculum conducted by female physicians [1]	0	506	0.0 (0.0, 7.3)
Al-Riyami et al., 2007 [28]	Oman, Mixed	National Health Survey 2000 aiming to identify reproductive morbidity. Multi-stage stratified probability-sampling design of 1,968 households with ever married, non-pregnant women	Pelvic examination by a trained physician [1]	0	1,662	0.0 (0.0, 2.2)
Al-Qutob, 2001 [26]	Ain Al-Basha, Jordan. Semi-urban	Random sample of Jordanian women	Comprehensive physical and pelvic examination conducted by trained female physician, a nurse/midwife and a laboratory technician [1]	0	379	0.0 (0.0, 9.7)
Bulut et al., 1995 [25]	City of Istanbul, Turkey	Systematic sample of non-pregnant, ever married parous women who had ever used contraception	Physical examination by female physician [1]	0	696	0.0 (0.0, 5.3)
Tehrani et al., 2011 [34]	Four provinces of Iran	Multi-stage stratified probability-sampling design of non-pregnant non menopausal women 18-45	Comprehensive gynaecological examination of all married women including a speculum examination [1]	0	1117	0.0 (0.0. 3.3)
Studies with hospital based recruitment						
Ijaiya and Aboyeji, 2004 [23]	Ilorin, Nigeria, urban	Hospital review of women with fistula repair	Repair	34	32,188	1.1 (0.7, 1.5)
Kalilani-Phiri et al., 2010 [21]	Nine districts (urban and rural) in Malawi	Hospital record reviews from gynaecological, prenatal, obstetric wards and operating theatres as well as fistula repair services. Only women originating from nine districts included	Repair	111	425,865	0.26 (0.2, 0.3)
Mabeya, 2004 [36]	West Pokot, Kenya. Rural	Hospital record review supplemented by surgeons’ notes. Cases of fistulae presenting to the two rural hospitals that are the main hospitals in the district	Repair	66	150,000	0.44 (0.34, 0.55)

[1] These studies were reproductive morbidity studies which did not state in the methods that they were investigating fistula, nor did they report any cases of fistula; however the type of examination used to identify other reproductive morbidities was assessed to have been sufficient that should there have been any cases of fistula they would have been identified.

**Table 2:** Characteristics of studies reporting fistula incidence included in the review

**Author**	**Study area**	**Study design**	**Assessment of fistula**	**Number of fistula**	**Number of women/pregnancies**	**Incidence(per 1000 pregnant women)**
Community based studies						
Vangeenderhuysen et al., 2001 [22]	Eight centres (urban and rural) in six countries in West Africa	Prospective cohort study of all pregnant women found by a door to door census of households followed up from antepartum to two months postpartum.	Women reporting gynaecological problems. Fistula assessed at last contact 60 days after delivery	2	19,694	0.10 (0.01, 0.3)
Ferdous et al., 2012 [33]	Matlab, Bangladesh. Rural	Prospective cohort of all women with obstetric complications, a perinatal death or caesarean section, and random sample of women with uncomplicated births.	Physical examination at health centre from six to nine weeks postpartum	0	1,162	0 (0, 3.17)
Fronczak et al., 2005 [20]	Urban slums in Dhaka, Bangladesh	Prospective community-based study of women completing at least seven months of pregnancy. Women excluded if birth identified more than 21 days postpartum. Sample selected using multi-stage probability.	Physical exam conducted by female physicians conducted one-month postpartum	0	557	0 (0.0. 6.6)
Studies with hospital based recruitment						
Filippi et al., 2007 [30]	Seven public urban and rural hospitals in Burkina Faso	All women with severe obstetric complications delivering in hospitals and two controls per case. Interviews conducted at 3, 6, and 12 months after pregnancy.	Medical examinations	1	1,014	0.99 (0.03, 5.48)
Filippi et al., 2010 [31]	Cotonou, Porto Novo and neighbouring communities in south Benin	Prospective cohort study of women with severe obstetric complications and a sample of women with uncomplicated childbirth.	Medical examination with obstetricians	1	709	1.41 (0.04, 7.83)
Prual et al., 1998 [32]	Niamey city, Niger. Urban	All deliveries in six maternity wards, and all complications referred to the two referral maternity wards occurring from 28^th^ week antenatal to 42^nd^ day postpartum.	Medical examinations	2	4,081	0.49 (0.06, 1.77)

Three community-based studies were from sub-Saharan Africa [18,22,24], four were from South Asia [19,20,33,35], two were from North Africa [28,29], three from the Middle East [26,27,34], and one from Turkey [25]. All six studies using hospital-based recruitment were from sub-Saharan Africa [21,23,30-32,36].

**Figure 3:**
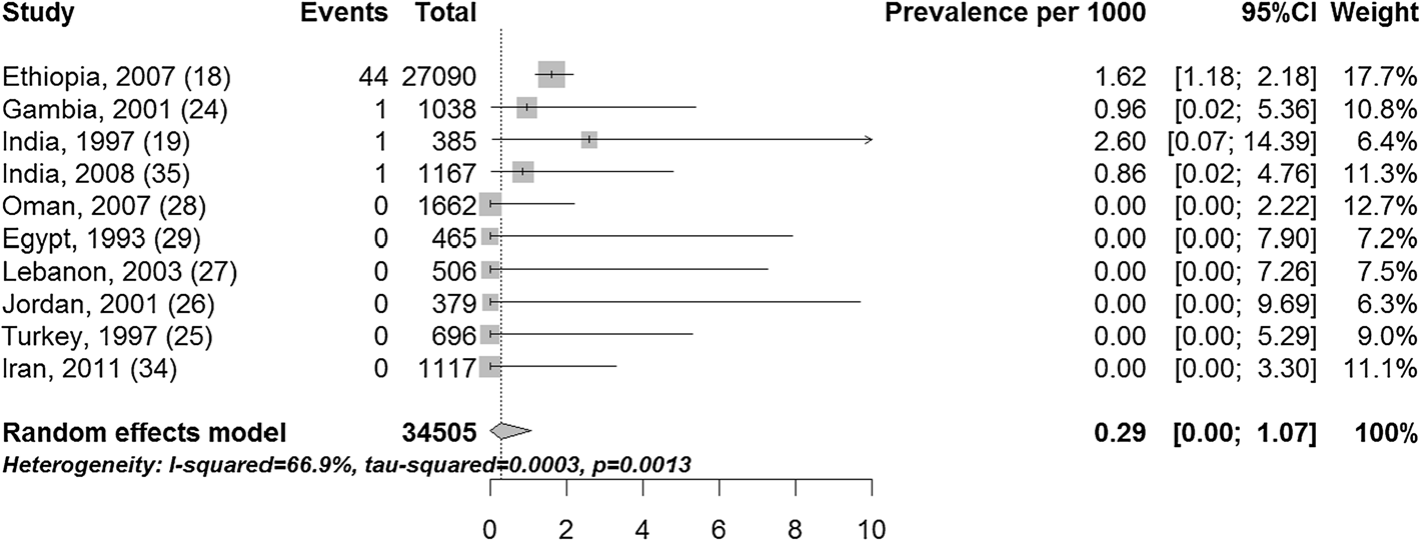
Prevalence of fistula per 1000 women of reproductive age.

**Figure 4:**
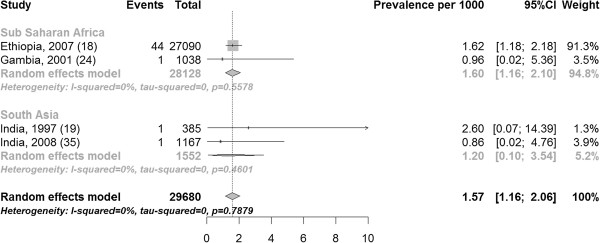
Prevalence of fistula per 1000 women of reproductive age stratified by region.

The prevalence of fistula in community-based studies ranged from 0 to 1.62 per 1000 women of reproductive age with a median of 0 per 1000 (Figure 3). The pooled prevalence of fistula in community-based studies was 0.29 (10 studies including 34,505 participants 95% CI 0.00, 1.07) per 1000 women of reproductive age (Figure 3). The pooled estimate for sub-Saharan Africa and South Asia was 1.13 (4 studies with 29,680 participants 95% CI 0.72, 1.61) per 1000 women of reproductive age (Figure 4). By continent Sub-Saharan Africa had an overall prevalence of 1.60 (two studies including 28,128 participants 95% CI 1.16, 2.10) per 1000 women of reproductive age and South Asia had a prevalence of 1.20 (two studies with 1552 participants 95% CI 0.10, 3.63) per 1000 women of reproductive age (Figure 4). Because all studies from the Middle-East and North Africa had zero events, it is impossible for the meta-analysis to provide a meaningful estimate due to the manner with which zero prevalence studies are dealt with (http://cran.r-project.org/web/packages/meta/meta.pdf).

**Figure 5:**
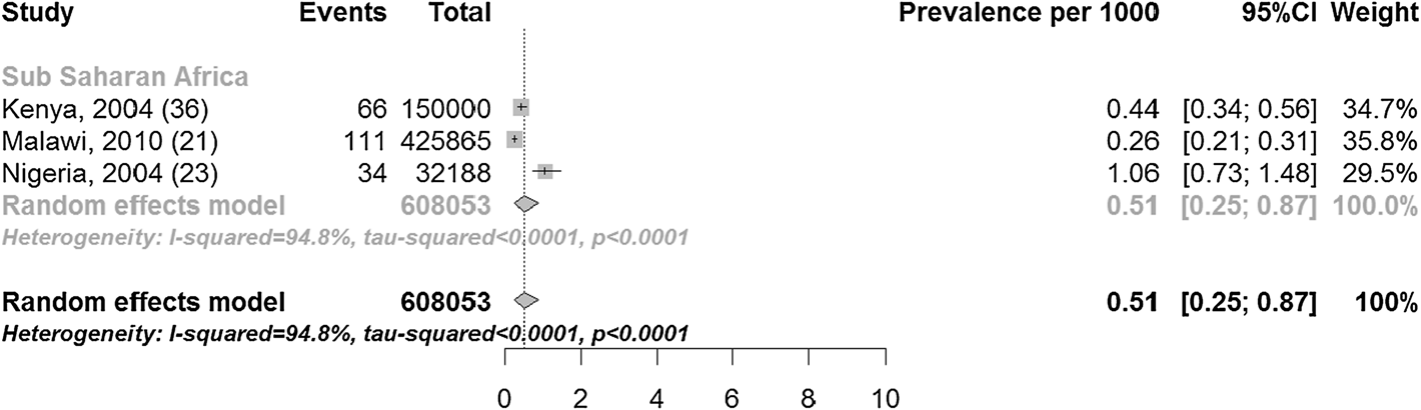
Prevalence of fistula per 1000 women of reproductive age in studies with hospital-based recruitment.

The prevalence of fistula in studies with hospital-based recruitment ranged from 0.26 to 1.06 per 1000 women of reproductive age, with a median of 0.44 per 1000 women of reproductive age. The pooled estimate of the prevalence of fistula in hospital was 0.51 (three studies with 608,053 participants 95% CI 0.25, 0.87) per 1000 women of reproductive age (Figure 5).

**Figure 6:**
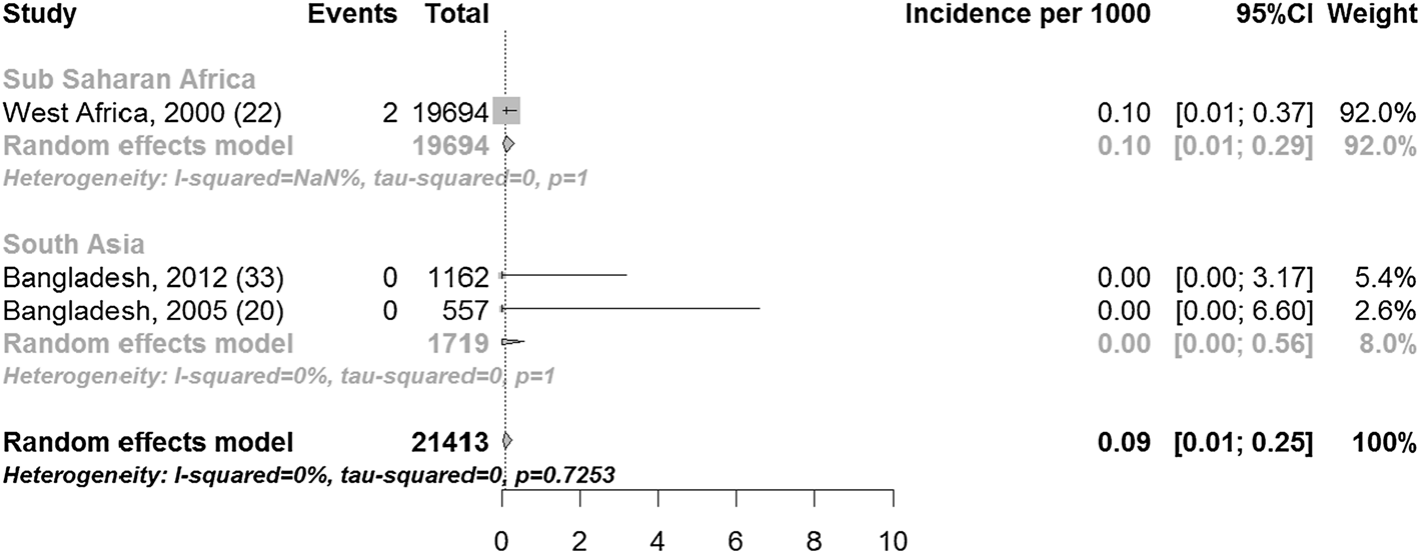
Incidence of fistula per 1000 pregnancies in community-based studies, stratified by region.

**Figure 7:**
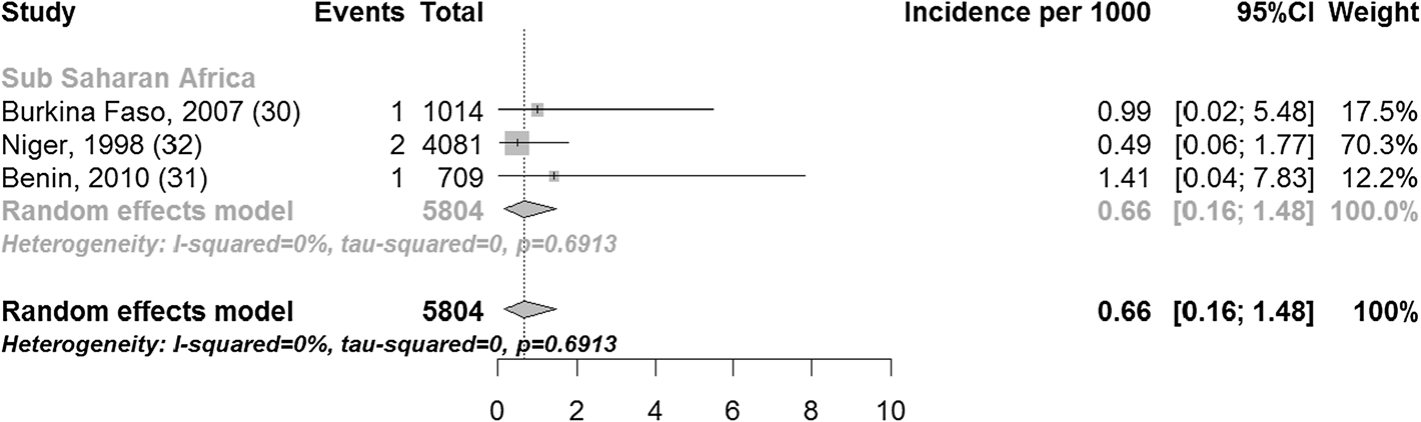
Incidence of fistula per 1000 pregnant women in studies with hospital-based recruitment.

The pooled incidence of fistula in community-based studies was 0.09 (three studies with 21,413 participants 0.01, 0.25) per 1000 recently pregnant women (Figure 6) and in hospital-based studies 0.66 (three studies including 5804 participants 0.16, 1.48) per 1000 recently pregnant women (Figure 7).

The I [2] values varied substantially by stratum, ranging from 0% in community-based (Figure 6) and hospital-based (Figure 7) incidence studies to 94.8% in prevalence studies with hospital based recruitment (Figure 5). Study quality in the modified Ottawa-Newcastle score table is shown in Table 3. All studies had a cross-sectional or cohort design.

**Table 3:** Sources of risk of bias in included studies

	**Selection**			**Comparability**	**Outcome**		
**Study:**	**What is the case definition? (Condition of interest)**	**Representativeness of the study population**	**Selection of non cases**	**Comparability of cases and non-cases**	**Assessment of outcome**	**Was study long enough to ensure cases would be found**	**Differential follow up?**
Muleta et al., 2008 [18]	Obstetric fistula treated and untreated	Population based sample of seven administrative regions of rural Ethiopia	Only women reporting leaking examined, therefore it is possible that some women may have been counted as non cases	All from same population	Sufficient physical exam	As sample was women of reproductive age, some women will have only just given birth and it is possible they may have not yet developed fistula	Do not state
Walraven et al., 2001 [24]	Obstetric morbidities including fistula	Population based rural region	All women invited for a physical examination	All from same population	Sufficient physical exam	As sample was women of reproductive age, some women will have only just given birth and it is possible they may have not yet developed fistula	28% of sample did not have examination
Kulkarni, 2007 [35]	Obstetric morbidities including fistula	Population based	Included all women examined	All from same population	Sufficient physical exam	Women with children at least six months examined so assumption is that it would be six months postpartum	25% of sample women did not have examination
Bhatia et al., 1997 [19]	Gynecological morbidity including fistula	Population based	Included all women examined	All from same population	Sufficient physical exam	Women had exam after one year so long enough for fistula to develop	5% lost to follow up, 6% not examined
Younis et al., 1993 [29]	Gynaecological and related morbidities, but do not state that they looked for fistula	Population based	Included all women examined	No cases	Sufficient physical exam	Women who were ever married and not pregnant, so it is possible they may have not yet developed fistula	Do not state
Deeb et al., 2003 [27]	Gynaecological and related morbidities, but do not state that they looked for fistula	Population based	Included all women examined	No cases	Sufficient physical exam	Women who were ever married and not pregnant, so it is possible they may have not yet developed fistula	9% did not have examination
Al-Riyami et al., 2007 [28]	Gynaecological and related morbidities, but do not state that they looked for fistula	Population based from national survey	Included all women examined	No cases	Sufficient physical exam	As sample was women of reproductive age, it is possible they may have not yet developed fistula	Do not state
Al-Qutob, 2001 [26]	Gynaecological and related morbidities, but do not state that they looked for fistula	Population based	Included all women examined	No cases	Sufficient physical exam	Women who were ever married and not pregnant, so it is possible they may have not yet developed fistula	10.7% did not have examination
Bulut et al., 1995 [25]	Gynaecological and related morbidities, but do not state that they looked for fistula	Population based, but in Istanbul which may not be representative of Turkey as a whole. Additionally only included women who had ever used contraception	Included all women examined	No cases	Sufficient physical exam	Unclear how long women were followed up for after pregnancy	5% did not have examination
Tehrani et al., 2011 [34]	Gynaecological and related morbidities, but do not state that they looked for fistula	Population based	Included all women examined	No cases	Sufficient physical exam	All women from 18-45 who were not pregnant, so it is possible some may not have had time for fistula to form	119 dropped out
Ijaiya and Aboyeji, 2004 [23]	Obstetric fistula	Hospital record review of fistula repairs with details about reference population	Case series of repairs	No non cases	Physical exam and treatment	All women already had fistula. Possible that women missed who did not present for treatment	N/A
Kalilani-Phiri et al., 2010 [21]	Obstetric fistula	Hospital record review with details of population it came from, however researchers eliminated all cases not originating in the region.	Case series of repairs	No non cases	Physical exam and treatment	All women already had fistula. Possible that women missed who did not present for treatment	N/A
Mabeya, 2004 [36]	Obstetric fistula	Hospital record review of fistula repairs with details about reference population	Case series of repairs	No non cases	Physical exam and treatment	All women already had fistula. Possible that women missed who did not present for treatment	N/A
Vangeenderhuysen et al., 2001 [22]	Obstetric morbidities including fistula	Population based	Included all women examined	All from same population	Sufficient physical exam	Followed up to 60 days after birth	5.7% loss to follow up
Ferdous et al., 2012 [33]	All short and long term postpartum morbidities including fistula	Women with morbidities and random sample of all women	Included all women examined	All from same population	Sufficient physical exam	Examined 6-9 weeks postpartum	4.1% lost to follow up and 6.1% did not have examination
Fronczak et al., 2005 [20]	Obstetric morbidities including fistula	Population based	All women examined, but women who may have had fistula followed up longer	All from same population	Sufficient physical exam	Women feared to have fistula followed up one month postpartum	63% did not have examination
Filippi et al., 2007 [30]	Severe obstetric complications including fistula	Women with complications over-represented but also had follow up of women with uncomplicated birth	All women examined	All from same population	Sufficient physical exam	Women had follow up at six months	11% only had either interview or physical exam at six months
Filippi et al., 2010 [31]	Severe obstetric complications including fistula	Women with complications over-represented but also had follow up of women with uncomplicated birth	All women examined	All from same population	Sufficient physical exam	Women had follow up at six months	32% of women did not have follow up at six months
Prual et al., 1998 [32]	Severe obstetric complications including fistula	Women with complications over-represented but also had follow up of women with uncomplicated birth	All women examined	All from same population	Sufficient physical exam	Unclear how long women were followed up for after pregnancy so it is possible they may have not yet developed fistula	Do not state

Only two studies reported information on duration of fistula: one found a median of eight years [18], and the other a median of three years [21]. No community based studies reported on the mode of delivery of the women, or on the cause of fistula.

## Discussion

Our comprehensive systematic review found that fewer than 1 per 1000 women of reproductive age in low and middle income countries suffer from fistula; this figure rises to 1.57 per 1000 when only data from sub-Saharan Africa and South Asia is used. The number of new cases of fistula ranged from 0.09 per 1000 recently pregnant women in community-based studies to 0.66 per 1000 pregnancies in hospital-based studies.

The WHO has suggested that over two million women, mostly from sub-Saharan African and Asian countries, have fistula [1,37]. Given an estimated population of 645 million women of reproductive age in sub-Saharan Africa and South Asia in 2010 (http://esa.un.org/wpp/unpp/p2k0data.asp), this would suggest that 3 per 1000 women of reproductive age have a fistula, which is considerably higher than our estimate for low and middle income countries. There were too few studies in our review to arrive at robust estimates of the prevalence of fistula by continent, but even the highest estimates for sub-Saharan Africa (1.62 per 1000 women of reproductive age in Ethiopia) or South Asia (2.6 per 1000 in India) fall well short of the WHO estimates. One study [22] estimated an incidence of 1.239 per 1000 deliveries in rural regions and used this to approximate 33,451 new fistula a year in Sub Saharan Africa. This was based on 2 fistulae found in the “rural region” in the MOMA study. Elsewhere they refer to these regions as “small towns” [38]. They then used the sample of 2 fistulae per 1543 live births to estimate the overall incidence for sub-Saharan Africa. This approach is likely to have over-estimated the number of fistulae patients. First because a substantial proportion of African women now live in cities where the risk of fistulae is likely to be lower than in smaller towns or rural areas; second because the denominator does not include the urban women in larger cities for whom there were no fistulae occurrence.

Overall, we estimate that just over one million women may have a fistula in sub-Saharan Africa and South Asia, and that there are over 6000 new cases per year in these two world regions. Given the devastating consequences of fistula for women and their families, this represents a very substantial burden.

It is possible that community based studies represent an underestimate of the prevalence of fistula, as fistulae are generally more commonly found in regions where there is no access to obstetric care, and may be difficult to reach. These women may have been missed in the studies included here, and the estimates provided here may represent a lower bound estimate of prevalence. However both the studies in Ethiopia [18] and the Gambia [24] were conducted in rural areas, and both studies showed very low estimates of prevalence (1.62 and 0.96 respectively).

Our search identified 13 additional studies that would not have been found had the search been restricted to fistula only. This was particularly relevant for community-based prevalence studies, where the specific fistula search only identified four studies, compared to nine when the search was expanded to maternal and reproductive morbidity studies. Inclusion of studies that did not mention fistula could bias the results downwards since it is not certain that women were examined for fistula. However, our strategy of including only studies for which a thorough gynaecological examination was conducted should have ensured that fistula would have been diagnosed and reported if present. Additionally, we only included studies which elicited uterine prolapse, since this implied a thorough examination. Because fistula is a rare condition, it is important to take account of studies that did not find any fistulae, in the same way that studies with negative findings are crucial in systematic reviews of treatment effectiveness [39].

We were unable to draw solid conclusions about regional variations in the prevalence or incidence of fistula. We only found three community based studies from sub-Saharan Africa and four community-based studies from South Asia (all from India or Bangladesh). Many studies were from very select communities and it is uncertain whether these findings are generalisable to Africa and Asia as a whole. The heterogeneity in prevalence or incidence of fistula in community-based studies was very low however, suggesting that the prevalence and incidence of fistula was uniformly low across all study sites.

It is difficult to estimate the duration of fistula from the reviewed studies because only two papers provided an estimate. However, given that one of these from Malawi had a median duration of three years [21], and the other from Ethiopia [18] had a median duration of eight years, it seems that women can live with this condition for a very long time, in some contexts. Other studies have shown that women live with these conditions for years before presenting for fistula repair, sometimes as long as over 20 years [10]. Additionally there was a lack of information on the mode of delivery and cause of the fistula in the community based studies, meaning that it is possible that some women suffered a fistula from causes other than prolonged or obstructed labour.

The low prevalence and incidence of fistula among women recruited in hospital is somewhat unexpected, since women seeking care from hospitals tend to be self-selecting because they are ill. The low incidence among women followed in the community after admission to hospital with near-miss obstetric morbidity (0.9 per 1000 and 1.4 per 1000) [30,31] is particularly surprising, since this sample would have included a substantial number of women with a prolonged and complicated labour. For example in Benin 27.1% of women had near-miss due to dystocia, which would have included women with both obstructed and prolonged labour [31]. Because these women were recruited in hospital, a timely caesarean section may have prevented the fistula from developing. Reduced fertility is common in women with fistula (due to loss of vagina, amenorrhoea, not engaging in intercourse, and inability to have a live baby) [2,7,9], and it is unlikely that women in the included incidence studies would have had a fistula before getting pregnant. Attention should be drawn to the fact that the meta-analysis included one study from Ethiopia that had 76.4% (Figure 3) or 91.3% of the weight in community-based studies (Figure 4), possibly inflating the pooled prevalence estimate.

The low frequency of fistulae in any community make survey enquiries that specifically target the counting of fistula cases prohibitively expensive given the large sample sizes required. Still, all community based studies of reproductive health should explicitly ascertain and report the clinical presence or absence of fistula, even when the sample size is small. .

In many countries in sub-Saharan Africa there is an emphasis on building specialised fistula hospitals dedicated to the treatment of women suffering from fistula. Given the rarity of the condition and the high level of skills and training required for fistulae surgery, the results of this review suggest that the majority of the resources will always be better placed on prevention rather than cure. Strengthening maternal health services, creating conditions for better transportation and communication networks and training of local providers into the management of emergency complications, including with caesarean sections, would have the additional effect of providing care for other causes of maternal and perinatal mortality and morbidity.

The relative rarity of fistula should not detract from their public health importance. The estimated 6000 new cases of fistula per year in sub-Saharan Africa and South Asia are a painful testament to the continued failure of health systems to manage labour complications effectively. Caesarean sections remain inaccessible to a large number of women in sub-Saharan Africa [40]. Delays in accessing caesarean sections, faulty techniques and lack of caesarean sections all contribute to the burden of fistula. The fact that fistula have virtually disappeared in high income countries suggest that they are entirely preventable. Given the seriousness of the condition, and the devastating consequences of fistula for women and their families, efforts should also be made to find these women and treat them.

## Conclusions

Our study is the most comprehensive study of the burden of fistula to date, including study sources not generally used. Our findings suggest that the prevalence and incidence of fistula is relatively low. The low burden of fistula should not detract from their public health importance, however, given the preventability of the condition, and the devastating consequences of fistula. Future studies of fistula should include a description of the study population with defined denominators.

## Competing interests

The author’s declare that they have no competing interests.

## Authors’ contributions

AJA conducted search, extracted data, did analysis and wrote first draft of paper CR advised on methodology and commented on drafts CC commented on drafts and helped in writing of paper VF helped design study and commented on paper. All authors read and approved the final manuscript.

## Pre-publication history

The pre-publication history for this paper can be accessed here:

http://www.biomedcentral.com/1471-2393/13/246/prepub
